# Perfectionism and anxiety sensitivity in soccer referees: the mediating role of distress tolerance

**DOI:** 10.3389/fspor.2026.1914085

**Published:** 2026-08-26

**Authors:** J. J. Martínez-Oller, A. P. Pacheco-Unguetti, J. González-Hernández

**Affiliations:** Sport, Health and Performance Psychology Lab; Mind, Brain, and Behavior Research Center (CIMCYC), University of Granada, Granada, Spain

**Keywords:** anxiety sensitivity, distress tolerance, mental health, perfectionism, referees

## Abstract

**Background:**

The present research aimed to analyze the relationship between perfectionism, sensitivity to anxiety, and distress tolerance in soccer referees.

**Methods:**

The sample consisted of 268 Spanish soccer referees from different categories and experience levels, and a correlational and cross-sectional design was used. Validated instruments such as the *Multidimensional Perfectionism Scale – Short Form* (MPS-SF), the *Anxiety Sensitivity Index-3* (ASI-3), and the *Distress Tolerance Scale* (DTS) were employed.

**Results:**

The results showed that perfectionism, especially socially prescribed perfectionism, co-occurred with higher anxiety sensitivity, thereby increasing referees’ emotional burden. On the other hand, referees with higher distress tolerance showed a better manage of their emotions better under pressure, which positively impacted their performance perceived and decision-making. In addition, distress tolerance partially mediated the relationship between perfectionism and anxiety sensitivity.

**Discussion:**

In conclusion, the implementation of psychological training programs is essential to improve referees’ distress tolerance and emotional coping strategies, optimizing both their performance and psychological well-being in the professional domain.

## Introduction

Refereeing in general—and soccer refereeing in particular—is a highly demanding activity that requires physical, cognitive, and emotional skills. From the early stages in youth leagues (even as teenagers) through to the professional level, referees are exposed to constant pressure, both external (e.g., players, coaching staff, spectators) and internal (e.g., self-perceived competence, fear of making mistakes) (1). The constant evaluation and public scrutiny of their performance, especially with the implementation of technologies such as the Video Assistant Referee (VAR) (2, 3), have intensified this exposure, as well as the levels of psychological vulnerability experienced by these professionals (4–6).

In Spain, for example, incidents of violence are constantly reported in grassroots sports and, on a larger scale, in many televised matches. Similarly, in professional refereeing (i.e., Championship World, Continentals Tournaments, Champions Clubs League, National Clubs Leagues), cases of harassment and constant exposure to criticism are reported on a daily basis, contributing to a highly emotionally demanding environment that creates an intimidating atmosphere which negatively impacts referees’ confidence and development from the very start of their training (7, 8).

It is therefore necessary to identify personal factors that may facilitate and/or hinder the development of coping strategies. These strategies are essential for maintaining focus and self-control, as well as to demonstrate and uphold impeccable ethics and impartiality as fundamental principles, basing every decision strictly on the rules and remaining free from external influences during high-pressure situations. Historically subject to scrutiny and viewed as impersonal, their decisions go beyond the purely athletic realm.

To address these challenges, mental toughness and a focus on the task at hand, without being swayed by external factors, are considered essential traits of successful referees (9, 10). In this context, the ability to make objective decisions, together with strong self-confidence, is key to performing this role effectively and sustainably over time (11).

### At what point did refereeing become a “psychologically demanding activity"?

Today, soccer has transcended its sporting essence to become a global spectacle in which media pressure, fanaticism, and economic interests have substantially altered the perception of refereeing (12). Examples of these pressures include decisive penalty decisions, offside calls, dismissals of players or coaches that alter the course of a match, officiating errors widely publicized through traditional and social media, public criticism from supporters, statements by coaches or club officials questioning referees’ impartiality or competence, and threats or harassment on social media platforms. In this context, referees must not only manage the pressure of the game but also the impact of decisions that can influence multimillion-dollar economic interests (e.g., player transfers, betting). This significantly increases their exposure to stressors, rumination, and the “inevitability” of non-acceptance of the ruling (13).

Amateur leagues also exhibit high rates of negative behavior toward referees (14, 15), prompting concerns regarding potential aggression due to closer proximity to spectators and the younger age of the officials (16). Furthermore, amateur referees possess fewer resources to execute their duties. Consequently, lower-league match officials may be more vulnerable to mental health issues than their elite-level counterparts (17, 18).

### Identifying some psychological needs in the role of the referee

To maintain consistency with the pace of the game, referees must rely not only on adequate physical conditioning (19) but also on advanced cognitive abilities such as mental flexibility (20), rapid and accurate decision-making (21), moral judgment (22, 23), or effective communication with their assistants, players, and other members of the team (24, 25).

Aspects such as resilience, staying mindful and focused on every moment and detail of the game, and coping with stress are therefore essential for a referee, who is also exposed to constant criticism and conflict (where high levels of rejection and aggression are perceived) (20). Despite these demands, they receive little or no psychological training and/or preparation (26, 27).

A qualitative study conducted with FIFA referees found that self-control, the ability to concentrate, and emotional regulation are key factors in refereeing performance. The study concluded that referees with a greater ability to manage anxiety and tolerate uncertainty were significantly more effective when making decisions under pressure (6).

The need and desire to maintain flawless performance, free from criticism and controversy, foster a drive toward perfection characterized by high intensity and psychological strain (28, 29), has been identified with the pursuit of perfection. The Multidimensional Perfectionism Model [MPS; (29–31)] describes the hierarchical functioning of perfectionism (level 1: traits; level 2: cognitions; and level 3: self-presentations), taking into account both interpersonal and intrapersonal interaction styles. To Hewitt & Flett, perfectionism is characterized by the pursuit of extremely high standards, excessive self-criticism in the face of mistakes, and efforts to avoid imperfections in front of others. As traits (see Table 1), the MPS distinguishes between self-oriented (e.g., set high standards for oneself, place great value on them, and make significant efforts to achieve them), socially prescribed (e.g., to interpret, in looking for the social approval, that the contexts in which a person interacts entail a set of expectations that must be met, depending on the role they play), and other-oriented (e.g., to expect those around them to be perfect, and also being highly critical of their mistakes).

**Table 1:** Examples of perfectionist thoughts as they apply to referees (adapted by the authors).

Trait	Common cognitions among the general population	Common cognitions among the referees
Self-oriented (SOP)	*“Everything has to go perfectly for me”*	*“This game has to go perfectly for me”, “I have to do my very best not to make any mistakes”*
Other-oriented (OOP)	*“Everyone else should do things perfectly”*	*“The officials have to do a perfect job for the game to go smoothly”*
Socially prescribed (SPP)	*“Everyone else expects me to do everything perfectly”*	*“Everyone in the stands who came to the game is hoping I’ll do everything perfectly”*

In the context of refereeing, where referees are constantly subject to external judgment and scrutiny regarding what is expected of them, socially prescribed perfectionism takes on particular significance. Recent research has linked perfectionism to the activation of the behavioral inhibition system, which leads to hypersensitivity to negative feedback and promotes rumination (a repetitive cognitive process focused on negative past events) regarding mistakes and criticism received (32, 33). Rumination has been identified as a mediating factor in the relationship between perfectionism and emotional distress (34, 35).

The psychological reactivity that arises from the interplay between the drive for perfection and the fear of failure is therefore a key psychological factor in refereeing, not only in soccer but in any sport. Studies on referees identify a relationship between perfectionism and the fear of making mistakes, highlighting that referees with a greater perfectionist tendency also tend to experience a greater fear of failure, which negatively impacts their performance and psychological well-being. In particular, negative reactions to imperfection such as excessive self-criticism, self-handicapping, or social disconnection, have been frequently reported (36–38).

Furthermore, a high level of perfectionism combined with heightened sensitivity to anxiety can lead to an overemphasis on mistakes and the avoidance of conflictual situations, which undermines their authority and confidence in decision-making (39). Likewise, a low distress tolerance can trigger impulsive reactions or hesitation at critical moments, affecting the flow of the game and increasing the likelihood of errors (40). In this regard, it has been observed that referees who receive training in emotional regulation and stress coping strategies demonstrate better performance and a lower incidence of emotional exhaustion (13).

Other psychological phenomena, such as the catastrophizing of pain (especially when dealing with the consequences of physical injuries) (41), psychological inflexibility (a tendency to avoid or refuse to accept difficult emotional experiences) (42), narcissistic attitudes (43), aggression and perceived (44), or challenge appraisals and psycho-biosocial experiences (38), have emerged as a growing focus of research on referees in recent years.

### Sensitivity to psychological distress and the need for internal regulation

In this context, *sensitivity to anxiety* (SA) plays a significant role, as referees—being individuals highly exposed to criticism—tend to perceive stressful situations as threats, thereby increasing their emotional reactivity and impairing decision making under pressure (13, 45).

Directly connected with perfectionistic patterns (46), referees’ emotional regulation could be influenced by anxiety sensitivity, defined as the fear of experiencing physiological symptoms of anxiety and the belief that these may have serious consequences (47–49). High levels of anxiety sensitivity have been linked to an increased risk of developing anxiety disorders and depression (50), and in athletes, it causes them to mistake normal bodily responses for threats, increasing their performance anxiety.

Extensive psychometric research establishes that the original Anxiety Sensitivity Index (ASI) possesses a hierarchical, multidimensional structure applicable across both adult and youth cohorts (51–53). This multidimensional framework can be very effective in capturing how sport people (e.g., athletes, referees, coaches) process autonomic arousal. Specifically, the ASI describes a singular higher-order factor—general anxiety sensitivity—underpinned by three distinct lower-order dimensions (53, 54). First, *physical concerns* involve a dread of somatic sensations, such as an athlete misinterpreting an elevated heart rate as a medical emergency rather than normal exertion. Second, *social concerns* entail the fear of publicly observable anxiety symptoms, exemplified by a referee worrying that visible trembling while making a critical call will invite crowd ridicule. Third, *cognitive concerns* reflect a fear of psychological dyscontrol, such as a coach fearing that racing thoughts under high pressure signify an imminent loss of mental clarity.

Previous research indicates that this condition exacerbates physical and cognitive symptoms during competition (45, 55). This reduces the athlete's concentration and negatively affects their performance. Furthermore, this trait heightens the perception of risk at critical moments and leads to ineffective responses under pressure. In soccer refereeing, this characteristic manifests as a greater susceptibility to pre-competitive stress, particularly in evaluative situations, such as physical tests and performance assessments (19).

When exploring which variables influence the affective prediction of anxiety states (56), *distress tolerance* (DT) emerges as a key factor. Defined as the ability to withstand and manage complex psychological states, based on how we evaluate our negative emotions across four dimensions: their aversiveness, their acceptability, their capacity to distract us and disrupt our routine, and emotional control (57, 58), distress tolerance may play a decisive role in stress management, the perception of pressure, and perfectionist traits in refereeing. It refers to referees’ ability to cope with and endure negative emotional states without resorting to avoidance strategies (59). Low distress tolerance has been associated with ineffective coping strategies, which can exacerbate anxiety and depressive symptoms in high-pressure contexts (60). Hence, the scientific literature has indicated that the presence of appropriate strategies that promote distress tolerance, emotional resilience, mental flexibility, and coping skills has proven effective in optimizing performance and preserving psychological well-being in both young athletes (39) and soccer referees (20).

### Aims and hypotheses

With the aim of answering the question: What psychological resources can be identified in referees that enable them to manage psychological distress during their performance, which is subject to the pressure to be perfect?, this study seeks to analyze the relationship between perfectionism, sensitivity to anxiety, and distress tolerance among soccer referees, assessing how these psychological variables influence their emotional regulation.

**Figure 1:**
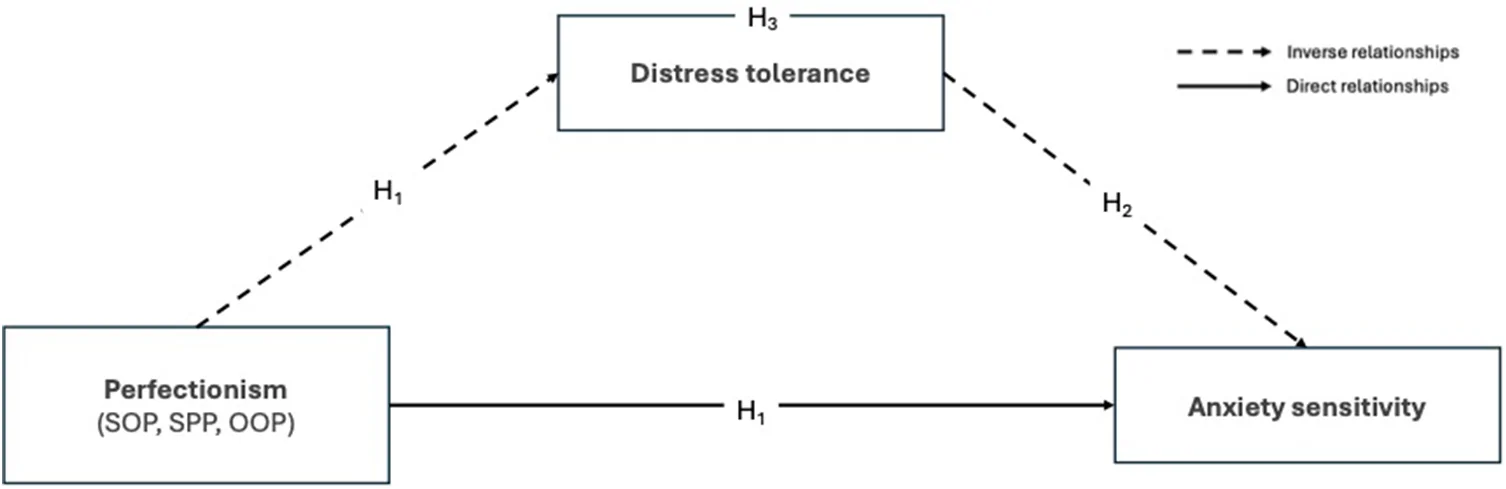
Hypothetical model.

To that end, and based on the scientific literature, the following hypotheses are proposed (see Figure 1): a) (H_1_) Socially prescribed and self-oriented perfectionism will be negatively associated with distress tolerance and positively associated with anxiety sensitivity; b) (H_2_) Distress tolerance will be negatively associated with anxiety sensitivity; that is, referees with higher levels of distress tolerance will exhibit lower levels of anxiety sensitivity, and; c) (H_3_) Distress tolerance will mediate the relationship between perfectionist traits and anxiety sensitivity in referees.

## Method

### Design

This study was conducted using a quantitative approach with a cross-sectional, correlational design, which allows for an objective analysis of the relationship between perfectionism dimensions, anxiety sensitivity, and distress tolerance in a sample of soccer referees from different divisions.

### Participants

A total of 268 Spanish soccer referees participated in the study, comprising 231 men (86.19%; *M* age = 26.24, *SD* = 9.32) and 37 women (13.81%; *M* age = 22.41, *SD* = 6.18). Some of them retired, but with > 20 years of experience as referees. Three experience levels were identified: 140 participants had fewer than 5 years of experience (80% men, 20% women), 75 reported between 5 and 15 years (89.33% men, 10.67% women), and 53 had more than 15 years (98.11% men, 1.89% women). Weekly training time also varied, with 45 referees training less than 3 h per week (80% men, 20% women), 128 training between 3 and 7 h (82.81% men, 17.19% women), 62 training between 7 and 10 h (91.94% men, 8.06% women), and 33 training more than 10 h per week (96.97% men, and 3.03% women). Additional sociodemographic and sport-related information is provided in Table 2.

**Table 2:** Sociodemographic and sport-related characteristics of referees by category.

Variable	Entry-Level (*n* = 41)	Regional (*n* = 166)	Semi-professional (*n* = 11)	Professional (*n* = 50)	Total (*N* = 268)	*p*
*Age*						**<.001**
Median (IQR)	18 (3.00)	21 (7.00)	26 (4.50)	34 (5.00)	23 (12.25)	
Mean (SD)	19.10 (4.90)	24.61 (9.03)	27.55 (3.27)	34.40 (5.51)	25.71 (9.04)	
Range	13–39	15–58	22–33	18–51	13–58	
*Gender, n (%)*						**.020** ^a^
Male	34 (82.93)	137 (82.53)^b^	11 (100.00)	49 (98.00)^b^	231 (86.19)	
Female	7 (17.07)	29 (17.47)^b^	0 (0.00)	1 (2.00)^b^	37 (13.81)	
*Education level, n (%)*						**<.001** ^a^
Basic–intermediate	29 (70.73)^b^	64 (38.56)	2 (18.18)	9 (18.00)^b^	104 (38.51)	
Undergraduate	12 (29.27)^b^	84 (50.60)^b^	3 (27.27)	22 (44.00)	121 (45.15)	
Postgraduate	0 (0.00)^b^	18 (10.84)^b^	6 (54.55)^b^	19 (38.00)^b^	43 (16.04)	
*Weekly training time n (%)*						**<.001** ^a^
<3 h	9 (21.95)	35 (21.08)^b^	1 (9.09)	0 (0.00)^b^	45 (16.79)	
3–7 h	20 (48.78)	98 (59.04)^b^	5 (45.45)	5 (10.00)^b^	128 (47.76)	
7–10 h	9 (21.95)	25 (15.06)^b^	3 (27.27)	25 (50.00)^b^	62 (23.13)	
>10 h	3 (7.32)	8 (4.82)^b^	2 (18.18)	20 (40.00)^b^	33 (12.31)	
*Years of experience n (%)*						**<.001** ^a^
<5 years	40 (97.56)^b^	100 (60.24)^b^	0 (0.00)^b^	0 (0.00)^b^	140 (52.24)	
5–15 years	1 (2.44)^b^	48 (28.92)	9 (81.82)^b^	17 (34.00)	75 (27.99)	
>15 years	0 (0.00)^b^	18 (10.84)^b^	2 (18.18)	33 (66.00)^b^	53 (19.78)	

*P*-value for age was obtained with Kruskal–Wallis test.

Bold *p*-values indicate statistically significant differences (*p* < .05).

a *P*-value corresponds to the Likelihood Ratio Chi-square tests (low expected frequencies are <5 in <20% of cells).

b Significant difference based on adjusted standardized residuals.

### Assessment tools

Participants first completed information regarding age, gender, educational level, refereeing category, years of experience, and weekly training hours.

#### Perfectionism

The Spanish version of the Multidimensional Perfectionism Scale – Short Form (61), adapted by Vicent et al. (62), was administered. It consists of 9items, rated on a Likert scale ranging from 1 (Strongly Disagree) to 7 (Strongly Agree). The scale assesses three dimensions: a) self-oriented perfectionism (SOP), reflecting the tendency to impose extremely high performance standards on oneself; b) other-oriented perfectionism (OOP), which refers to the expectation that others will maintain high standards; and c) socially prescribed perfectionism (SPP), characterized by the perception that others expect perfect performance. In the present sample, internal consistency for the scale dimensions was acceptable, with Cronbach's alpha values of .817 (SOP), .718 (OOP), and .782 (SPP).

#### Anxiety sensitivity

To measure anxiety sensitivity, the Anxiety Sensitivity Index–3 Scale (63) was administered. This is a revised version designed to assess anxiety sensitivity, defined as the fear of experiencing anxiety symptoms and their consequences. Adapted into Spanish by Sandín et al. (64), it consists of 18 items distributed across three subscales that include sensitivity to physical symptoms (e.g., fear of bodily manifestations of anxiety such as palpitations or shortness of breath), sensitivity to cognitive anxiety (e.g., fear of losing control or experiencing intrusive thoughts, rumination), and sensitivity to social anxiety (e.g., fear that others will notice anxiety symptoms and judge them negatively). The psychometric structure of the scale also accounts for a second-order global factor, termed the Anxiety Sensitivity Index (ASI), which will be the focus of this study. In the present sample, internal consistency was adequate to excellent for Physical Concerns (*α* = .838), Cognitive Concerns (*α* = .813), Social Concerns (*α* = .749), and the total score (*α* = .909).

#### Distress tolerance

To measure distress tolerance, the Spanish version of the Distress Tolerance Scale (DTS; 58), developed by Sandín et al. (65), was administered. This scale consists of 15 items, such as “*Feeling distress or discomfort is unbearable for me”*, to which respondents answer using a Likert scale with five response options, ranging from 1-“Strongly agree” to 5-“Strongly disagree”. The DTS assesses four subdimensions of distress tolerance: Tolerance, which refers to the perceived ability to withstand negative emotional states; Appraisal, which reflects the subjective evaluation of distress as acceptable or manageable; Absorption, which refers to the extent to which distress captures attention and interferences with functioning; and Regulation, which assesses the tendency to quickly reduce, avoid, or scape from distressing emotional experiences. In the present sample, internal consistency was adequate to excellent for Tolerance (*α* = .819), Appraisal (*α* = .755), Absorption (*α* = .833), Regulation (*α* = .794), and the total score (*α* = .905).

#### Procedure

The study procedure consisted of several phases that ensured proper data collection and compliance with the ethical and methodological criteria established for the research. During the recruitment phase, communication was established with refereeing bodies and sports associations, as well as with various individuals within the refereeing community—such as referees of different categories or informants—to inform potential participants about the study's objectives and scope. Interested referees were provided with detailed information explaining the methodological aspects of the research, data confidentiality, and their right to withdraw at any time without consequences. Before proceeding with data collection, participants were required to read an informed consent form guaranteeing the protection of their identity and the exclusive use of the information for academic and scientific purposes (https://forms.gle/PF8yyM3FUNBodQqf8). To ensure strict adherence to ethical research standards, the protection of anonymity and confidentiality, as well as voluntary participation and a clear description of the research objectives, the standards of the Declaration of Helsinki (66, 67) and the Ethical Committee of the University of Granada (2902/CEIH/2022) were followed.

### Data analysis

Statistical analyses were performed using JASP version 0.95.2 (68). The analyses followed a structured approach. First, descriptive analyses were conducted to characterize the sample in terms of sociodemographic and sport-related variables. Given that most variables violated normality assumptions (Shapiro–Wilk test), continuous variables were therefore described using medians and interquartile ranges (IQR), and group differences across refereeing categories were analyzed using the Kruskal–Wallis H test. For categorical variables, chi-square tests of independence or likelihood ratio chi-square tests (when expected frequencies were < 5) were used. Adjusted standardized residuals were examined to interpret significant associations. A significance level of *α* = .05 was adopted for all analyses.

Second, bivariate associations among perfectionism dimensions (SOP, OOP and SPP), DT, and ASI were examined using Spearman's rho correlations, given the non-normal distribution of most study variables. Third, a hierarchical linear regression analysis was conducted to examine the contribution of sociodemographic, sport-related and psychological variables to anxiety sensitivity. Predictors were entered in blocks according to the theoretical model, and model comparison was based on changes in explained variance (*Δ*R^2^), *F-*change and information criteria (AIC and BIC values). Finally, three independent simple mediation analyses were conducted to test whether DT mediated the relationship between each perfectionism dimension and anxiety sensitivity. Indirect effects were estimated using bias-corrected bootstrap confidence intervals based on 5,000 bootstrap resamples.

## Results

### Sample characteristics

The sociodemographic and sport-related characteristics of the sample are presented in Table 2. The final sample included 268 football referees distributed across four competitive categories: entry-level referees (*n* = 41), regional referees (*n* = 166), semi-professional referees (*n* = 11), and professional referees (*n* = 50). Significant differences between categories were found for age, education level, weekly training time, and years of experience (all *ps* < .001), as well as for gender (*p* = .020).

*Age* differed significantly across categories. Entry-level referees showed the lowest mean age (*M* = 19.10, *SD* = 4.90), followed by regional referees (*M* = 24.61, *SD* = 9.03), semi-professional referees (*M* = 27.55, *SD* = 3.27), and professional referees (*M* = 34.40, *SD* = 5.51). The total sample had a mean age of 25.71 years (*SD* = 9.04), with an age range from 13 to 58 years.

*Gender* distribution also differed significantly across categories (*p* = .020). The total sample was predominantly male, with 231 men (86.19%) and 37 women (13.81%). Women were mainly represented in the entry-level and regional categories, whereas the semi-professional category included only male referees, and the professional category included only one woman.

*Education level* also differed across refereeing categories (*p* < .001). Overall, basic-intermediate education was significantly more frequent among entry-level referees (70.73%), undergraduate education was the most frequent in the regional category (50.60%), and postgraduate education was more common among semi-professional (54.55%) and professional referees (38%).

*Weekly training* time differed significantly across categories (*p* < .001). Training between 3 and 7 h per week was the most frequent range among entry-level referees (48.78%), regional (59.04%), and semi-professional referees (45.45%). In contrast, professional referees reported significantly higher training volumes, with 50% training between 7 and 10 h per week and 40% training more than 10 h per week.

Finally, *years of experience* also differed significantly across refereeing categories (*p* < .001). Specifically, most entry-level referees had less than 5 years of experience (97.56%), as did 60.24% of regional referees. In contrast, most semi-professional referees indicated between 5 and 15 years of experience (81.82%), whereas most professional referees pointed out more than 15 years of experience (66%).

### Descriptive statistics of study variables

Descriptive statistics for the study variables are presented in Table 2. Regarding perfectionism and considering the theoretical range of the scale (3–21 per subscale), SOP showed the highest mean (*M* = 12.84; *SD* = 4.15), scoring slightly above the midpoint of the subscale. In contrast, OOP and SPP showed identical and lower means (*M* = 9.99; *SD* = 3.87 and 4.57, respectively). For the Distress Tolerance (DT) dimensions, Appraisal showed the highest mean (*M* = 3.48; *SD* = 0.77), followed by Absorption (*M* = 3.40, *SD* = 1.02), Tolerance (*M* = 3.26; *SD* = 1.07), and Regulation (*M* = 2.72; *SD* = 0.99). The DT score was also near the midpoint (*M* = 3.21; *SD* = 0.80). Regarding the ASI (0–24 per subscale; 0–72 total), ASI_Social_ Concerns showed the highest mean (*M* = 7.14; *SD* = 4.17), followed by ASI_Cognitive_ (*M* = 4.96; *SD* = 4.13) and ASI_Physical_ (*M* = 4.65; *SD* = 4.29). All three subscales and the Total ASI score (*M* = 16.75; *SD* = 11.14) fell below their respective midpoints.

### Bivariate correlations

 Table 3 presents Spearman correlations among the study variables. Regarding the association between perfectionism and anxiety sensitivity, all perfectionism dimensions showed significant positive correlations with ASI. The strongest association was observed between SPP and Total ASI (*rs* = .368, *p* < .001), followed by SOP and Total ASI (*rs* = .309, *p* < .001), and OOP and Total ASI (*rs* = .245, *p* < .001). At the subscale level, SPP showed positive correlations with ASI_Cognitive_ (*rs* = .351, *p* < .001) and ASI_Social_ (*rs* = .344, *p* < .001). SOP was positively correlated with ASI_Social_ (*rs* = .304, *p* < .001) and ASI_Cognitive_ (*rs* = .283, *p* < .001).

**Table 3:** Spearman correlations among study variables.

Variable	M (SD)	Range	1	2	3	4	5	6	7	8	9	10	11	12
1. SOP	12.84 (4.15)	3–21	—											
2. OOP	9.99 (3.87)	3–20	.496***	—										
3. SPP	9.99 (4.57)	3–21	.436***	.469***	—									
4. Tolerance	3.26 (1.07)	1–5	−.183**	−.273***	−.227***	—								
5. Appraisal	3.48 (0.77)	1.50–5.00	−.182**	−.285***	−.172**	.610***	—							
6. Absorption	3.40 (1.02)	1–5	−.175	−.152	−.229*	.761***	.695***	—						
7. Regulation	2.72 (0.99)	1–5	−.277**	−.160	−.046	.510***	.441***	.480***	—					
8. Total distress tolerance (DT)	3.21 (0.80)	1.37–4.96	−.238***	−.306***	−.260***	.878***	.800***	.887***	.735***	—				
9. AS_Physical_	4.65 (4.29)	0–23	.228***	.159**	.286***	−.371***	−.384***	−.418***	−.234*	−.431***	—			
10. AS_Cognitive_	4.96 (4.13)	0–21	.283***	.238***	.351***	−.406***	−.431***	−.432***	−.328**	−.462***	.616***	—		
11. AS_Social_	7.14 (4.17)	0–22	.304***	.246***	.344***	−.331***	−.364***	−.378***	−.247*	−.383***	.660***	.783***	—	
12. ASI	16.75 (11.14)	0–61	.309***	.245***	.368***	−.419***	−.442***	−.464***	−.307**	−.482***	.847***	.891***	.914***	—

*M,* mean; *SD,* standard deviation; Range, observed range. *p < .05; **p < .01; ***p < .001.

SOP, Self-Oriented Perfectionism; OOP, Other-Oriented Perfectionism; SPP, Socially Prescribed Perfectionism; DT, Distress Tolerance Global; ASI-3, Anxiety Sensitivity Index-3.

In relation to distress tolerance and anxiety sensitivity, all correlations were significant and negative. The strongest negative correlation was found between DT and Total ASI (rs = −.482, *p* < .001), followed by DT and ASI_Cognitive_ (rs = −.462, *p* < .001), and DT and ASI_Physical_ (rs = −.431, *p* < .001). Among the distress tolerance subdimensions, Absorption showed the strongest negative correlation with ASI (rs = −.464, *p* < .001), followed by Appraisal (rs = −.442, *p* < .001), Tolerance (rs = −.419, *p* < .001), and Regulation (rs = −.307, *p* < .01).

Regarding the association between perfectionism and distress tolerance (DT), the strongest negative correlations were observed between OOP and Appraisal (rs = −.285, *p* < .001), SOP and Regulation (rs = −.277, *p* < .01), and OOP and Tolerance (rs = −.273, *p* < .001). Additionally, DT was significantly and negatively correlated with SOP, OOP or SPP (all ps < .001).

### Regression analysis

A hierarchical linear regression analysis was conducted to examine the contribution of psychological and sociodemographic/sport-related variables to the Anxiety Sensitivity Index (ASI). Predictors were entered in three stages: 1) Distress tolerance (DT); 2) perfectionism dimensions (SOP, OOP and SPP); and 3) age, refereeing category, education level, years of refereeing experience, and gender (see Table 4).

**Table 4:** Hierarchical regression models predicting anxiety sensitivity (ASI).

Predictor	B	SE	*β*	t	*p*	95% CI
*Model 1* R² = .237, adjusted R² = .234, *Δ*R² = .237, F change = 82.495, *p* < .001, AIC = 1985.203, BIC = 1995.976						
Intercept	38.406	2.458		15.627	<.001	[33.567, 43.245]
DT	−6.738	0.742	−0.487	−9.083	<.001	[−8.199, −5.277]
*Model 2* R² = .317, adjusted R² = .306, *Δ*R² = .080, F change = 10.273, *p* < .001, AIC = 1961.507, BIC = 1983.053						
Intercept	26.037	3.641		7.152	<.001	[18.869, 33.205]
DT	−5.721	0.748	−0.413	−7.644	<.001	[−7.195, −4.248]
SOP	0.428	0.166	0.160	2.581	.010	[0.102, 0.755]
OOP	−0.160	0.180	−0.056	−0.892	.373	[−0.514, 0.194]
SPP	0.521	0.146	0.214	3.567	<.001	[0.233, 0.809]
*Model 3* R² = .348, adjusted R² = .315, *Δ*R² = .031, F change = 1.348, *p* = .212, AIC = 1966.998, BIC = 2020.863						
Intercept	27.724	4.464		6.210	<.001	[18.933, 36.516]
DT	−5.317	0.768	−0.384	−6.927	<.001	[−6.829, −3.805]
SOP	0.381	0.167	0.142	2.277	.024	[0.052, 0.710]
OOP	−0.086	0.189	−0.030	−0.456	.649	[−0.458, 0.286]
SPP	0.459	0.151	0.188	3.048	.003	[0.162, 0.756]
Age	0.050	0.106	0.040	0.469	.639	[−0.159, 0.259]
Refereeing category: Regional	−2.345	1.753		−1.338	.182	[−5.797, 1.106]
Refereeing category: Semi-professional	−3.771	3.541		−1.065	.288	[−10.744, 3.202]
Refereeing category: Professional	−4.696	2.600		−1.806	.072	[−9.816, 0.423]
Education level: Undergraduate	−1.172	1.298		−0.903	.367	[−3.729, 1.385]
Education level: Postgraduate	0.010	1.921		0.005	.996	[−3.774, 3.794]
Years of refereeing experience: 10–15 years	−2.235	1.743		−1.282	.201	[−5.668, 1.198]
Years of refereeing experience: >15 years	−1.334	2.842		−0.469	.639	[−6.931, 4.263]
Gender: Female	0.864	1.722		0.502	.616	[−2.527, 4.255]

B = unstandardized regression coefficient; S.E. = standard error; *β* = standardized regression coefficient; CI = confidence interval. Standardized coefficients are reported only for continuous predictors. Reference categories were entry-level referees for refereeing category, basic-intermediate education for education level, <5 years for years of refereeing experience, and male for gender. DT = Total Distress Tolerance. R² = proportion of variance explained by the model. Adjusted R² = R² corrected for the number of predictors included in the model. *Δ*R² = change in explained variance after adding a new block of predictors. F-change = test of whether the increase in explained variance between models is statistically significant. AIC, Akaike Information Criterion. BIC, Bayesian Information Criterion. Lower AIC and BIC values indicate better relative fit when comparing models.

Model 1, which included only DT as a predictor, was statistically significant, *F*(1, 266) = 82.495, *p* < .001, accounting for 23.7% of the variance in anxiety sensitivity (R^2^ = .237). When perfectionism dimensions (SOP, OOP and SPP) were introduced in Model 2, that model remained significant and produced a statistically significant increase in the explained variance, *Δ*R^2^ = .080, *F*change (3, 263) = 10.273, *p* < .001, increasing the total explained variance to 31.7% (*R^2^* = .317). Finally, the incorporation of the sociodemographic and sport-related variables (age, refereeing category, education level, years of experience, and gender) in Model 3 did not produce a statistically significant increase in the explained variance, *Δ*R^2^ = .031, *F*change (9, 254) = 1.348, *p* = .212, although the overall model remained significant, *F*(13, 254) = 10.425, *p* < .001, and the total variance explained reached 34.8% (R^2^ = .348). Consistent with this pattern, Model 2 showed the best relative fit, as reflected by lower AIC and BIC values than Model 3.

In Model 2, Distress Tolerance (DT) was the strongest negative predictor of ASI (*β* *=* *−.413, t* *=* *−7.644, p* *<* *.001, 95% CI [−7.195, −4.248]). SPP was a significant positive predictor of Total ASIl (β* = .214, *t* = 3.567, *p* < .001, 95% CI [.233,.809]), followed by SOP (*β* = .160, *t* = 2.581, *p* = .010, 95% CI [.102,.755]). OOP was not a significant predictor (*β* *=* *−.056, t* *=* *−0.892, p* *=* *.373, 95% CI [−.514,.194]).* Therefore, in the model showing the best relative fit, DT, SPP, and SOP were the variables that significantly explained ASI, whereas OOP did not reach statistical significance. Overall, higher levels of SPP and SOP together with lower DT were associated with higher anxiety sensitivity.

### Mediation analysis

In order to examine the mechanisms through which perfectionism dimensions influences referees’ anxiety sensitivity, three independent simple mediation analyses were performed. In each model, DT was entered as the mediator and anxiety sensitivity (ASI) specified as outcome variable. Each dimension of perfectionism (SOP, OOP and SPP) was entered as a predictor in its respective model. Table 5 shows the direct, indirect and total effects of each mediation model.

**Table 5:** Mediation results for multidimensional perfectionism and anxiety sensitivity vía total distress tolerance.

Predictor	Pathway	Std. estimate (β)	SE	z	*p*	95% BC CI [lower, upper]	Mediation type
SOP	Direct effect	.224	0.046	4.860	<.001	[0.129, 0.311]	Partial
	Indirect effect	.108	0.029	3.761	<.001	[0.056, 0.169]	
	Total effect	.332	0.052	6.360	<.001	[0.224, 0.426]	
OOP	Direct effect	.108	0.065	1.644	.100	[−0.022, 0.236]	Indirect
	Indirect effect	.137	0.033	4.170	<.001	[0.078, 0.207]	
	Total effect	.244	0.068	3.572	<.001	[0.107, 0.374]	
SPP	Direct effect	.258	0.060	4.317	<.001	[0.141, 0.374]	Partial
	Indirect effect	.100	0.029	3.477	<.001	[0.051, 0.166]	
	Total effect	.358	0.057	6.310	<.001	[0.243, 0.465]	

*N* = 268. All reported values are standardized coefficients (*β*). SE, standard error; BC CI, bias-corrected confidence interval based on 5,000 bootstrap resamples. Total Distress Tolerance was specified as the mediator and ASI-3 Total as the outcome variable.

SOP, self-oriented perfectionism; OOP, other-oriented perfectionism; SPP, socially prescribed perfectionism.

The mediation analyses revealed significant indirect effects for all three perfectionism dimensions, with bias-corrected confidence intervals excluding zero. SOP and SPP exhibited partial mediation patterns, showing both significant direct (SOP: *β* = .224, *p* < .001; SPP: *β* = .258, *p* < .001) and indirect effects through DT (SOP: *β* = .108, 95% BC CI [.056, .169]; SPP: *β* = .100, 95% BC CI [.051, .166]). In contrast, OOP showed a significant indirect effect (*β* = .137, 95% BC CI [.078, .207]) while its direct effect did not reach statistical significance (*β* = .108, *p* = .100), indicating an indirect-only mediation pattern.

## Discussion

The objective of this study was to analyze the relationship between perfectionism, anxiety sensitivity, and stress tolerance among soccer referees, assessing how these psychological variables influence their emotional regulation and refereeing performance. The results obtained, both descriptive and inferential, offer a deep understanding of the emotional and psychological dynamics faced by referees and provide valuable insights for their training and well-being. Furthermore, they allow us to determine whether the proposed hypotheses are confirmed or rejected for the sample analyzed.

With regard to H_1_, measures of perfectionism such as SPP and OOP were negatively associated with distress tolerance and positively associated with anxiety sensitivity (Total ASI and AS_cognitive_, AS_physical_, and AS_social_) among soccer referees. In general, it is observed that perfectionism, especially socially prescribed perfectionism, is associated with greater sensitivity to anxiety, which is consistent with previous studies linking the need to meet external expectations with increased anxiety (39, 69). The significant interactions revealed that higher levels of self-directed and socially prescribed perfectionism were associated with greater increases in shame and guilt after a competitive loss, consistent with previous findings (70). Although perfectionism seems to be beneficial for ambitious athletes who want to be the best—since it helps them stay focused and committed to their future goals, performance, and personal growth—their ability to get along with others suffers due to a constant state of alertness that exacerbates their tendency to avoid negative emotions (e.g., anxiety, shame, guilt) (71, 72).

Distress tolerance, both in its overall dimension and in its subdimensions, showed negative correlations with anxiety sensitivity (AS_Cognitive_, AS_Physical_, and AS_Social_). As we expected and therefore accepted H_2_, observed the co-occurrence that referees with higher distress tolerance exhibited lower levels of anxiety sensitivity, as expected. Various studies have shown that individuals who exhibit more positive attitudes, coping strategies, and tenacity demonstrate greater resilience in the face of setbacks (73). Adopting a positive coping style can mitigate or prevent the adverse effects of setbacks, and even transform them into beneficial factors (45, 55). In contrast, adopting a negative coping style not only fails to neutralize the negative impact of setbacks but may, in fact, intensify it (74).

The results confirmed H_3_ regarding the mediating role of DT (primarily indirect effects) in the predictive power of perfectionist traits (SOP, SSP, and OOP) on ASI. However, the hypothesis could not be fully confirmed for direct effects. In this regard, the direct effects were significant for SOP and SPP, but not for OOP. These results are consistent with previous research suggesting that high distress tolerance facilitates emotional regulation, which improves individuals’ ability to make decisions under pressure (13, 75). Although persistent perfectionist efforts (SOP) may contribute to certain trends towards tolerance, as evidenced in other studies with athletes (none in referees), the perception of distress and exhaustion remains challenging to mitigate when individuals are subjected to high-intensity perfectionist patterns, particularly in cross-sectional and longitudinal research (39, 76).

Sports experience and psychological maturity play a key role in referees’ ability to regulate their emotions and develop greater emotional and behavioral resilience. Whether or not there has been prior experience in other sports-related roles (such as a player or coach) (36), or whether referees have had prior experience playing other sports (24), the limited scientific literature focused on referees suggests greater emotional resilience, which can lead to more effective coping with or resistance to criticism and stressful situations. Similarly, as the results of this study indicate, referees with less experience in officiating and no prior experience in other sports roles are more susceptible to anxiety, which highlights the importance and necessity of comprehensive training in emotional skills.

### Dyscomfort tolerance, emerging through the influence of other psychological strategies and skills

Whether in general populations or in sports contexts, the literature has consistently documented the relationships between perfectionism and anxiety responses or other forms of maladaptive behavior, viewing these as sources of distress (29, 77). In many of these studies, other variables have been included to explain buffering, protective, or mitigating effects on this distress, which have helped to understand and highlight the value of emotional regulation capacities and skills in stressful situations, despite increasing patterns of psychological vulnerability (78, 79).

Variables such as self-efficacy (80), mindfulness (81, 82), and self-compassion (83, 84) have helped explain the importance of regulating emotional distress associated with perfectionism in sports. These variables can certainly be integrated into research proposals focusing on referees, who are so subject to the need to achieve perfection in their performances—a pressure that amplifies their psychological reactivity to anxiety, thereby affecting their mental health and, with a high probability, their performance and execution.

### Limitations and future research lines

Some of the limitations of this study lie, first and foremost, in its cross-sectional design, which allows for the identification of associations but does not establish definitive causal relationships, as noted in the methodology; thus, it is not possible to determine whether perfectionism leads to ASI and how the DT can mediate, reducing it, between this disturbed relationship. Although the sample size was relevant, its selection via non-probabilistic snowball sampling limited the generalizability of the results (85). Furthermore, representativeness could be improved in some of the strata in the arbitration panels (e.g., semi-professional level).

Finally, data collection relied exclusively on self-report questionnaires, which may be subject to biases such as social desirability, despite the control measures implemented to minimize them (anonymity). Furthermore, the imbalance in the sample concerning the group of women and the group of semi-professionals makes it difficult to describe the potential implications for the interpretation and generalization of the results. In future research, it would be useful to employ mixed-methods approaches, combining quantitative measures with direct observations of referees’ behavior in real game situations, experimental tests (e.g., cognitive tasks) and qualitative research (e.g., interviews or focus groups) to further inform interpretations of different types of officiating roles in various contexts and levels of sport (e.g., referees, judges, officials). Explore the relevance of other factors such as the level of media exposure, causal attributions, and teams’ expectations of referees.

As research on perfectionism demonstrates, the best opportunity to assess the trajectory of these efforts or tendencies toward perfection lies in longitudinal studies. These studies can help clarify the negative connotations associated with perfectionism and highlight the need to identify them as early as possible in order to develop appropriate training and psychological preparation strategies, focusing not only on performance but also on well-being.

## Conclusions

These findings underscore the importance of integrating emotional training strategies into referee training programs to optimise referees’ performance and mental health. This study describes that perfectionism, anxiety sensitivity, and distress tolerance are key psychological variables that affect emotional regulation and the performance of soccer referees. The results support the hypothesis that referees with higher levels of perfectionist traits experience higher levels of anxiety—as confirmed by the extensive literature in both sports and non-sports contexts—which interferes with their perceptions of well-being (increase ASI). It would be important to explore whether, through experience gained over time or through appropriate psychological training, distress tolerance could emerge—as observed in the present study—as a crucial protective factor in reducing anxiety and stress, minimizing interference with decision-making and contributing to greater psychological well-being among referees.

Studies involving samples of referees (not limited to contexts such as soccer) are essential for gaining a deeper understanding of one of the most prominent roles in any sport (i.e., judges, commissioners). The scarcity of studies reflects, first, the heavy focus of sports psychology on athletes, but also the limited attention paid to those who increasingly influence outcomes and the application of rules, and who must also maintain balance and optimal psychological conditions for their own performance. Furthermore, given the pressure they face—particularly in high-profile sports or at the highest competitive levels—such psychological preparation is essential for them to reach their full performance potential and ensure their well-being.

Finally, given the small effect size, interventions should not focus exclusively on distress tolerance. Instead, it may be more effective to incorporate distress tolerance training (adapted to referees category and social pressure) within a broader psychological skills program that also addresses perfectionistic concerns, cognitive appraisal, emotional regulation, self-compassion, and coping with external evaluation and criticism [i.e., normalize mistakes and external criticism (interventions can help them reinterpret criticism and reduce the perception that they must meet others’ unrealistically high expectations); develop distress tolerance skills, including techniques from Acceptance and Commitment Therapy (ACT) or Dialectical Behavior Therapy (DBT); integrate these skills into referee education (rather than delivering standalone distress tolerance workshops, psychological skills could be embedded within existing referee development programs alongside attentional control, decision-making under pressure, and emotion regulation); or individualize interventions (screening for high levels of socially prescribed perfectionism could help identify those most likely to benefit)].

## Data Availability

This manuscript is part of the doctoral dissertation by Juan J. Martínez-Oller, a student in the doctoral program in Psychology at the University of Granada.

## Data availability statement

The datasets presented in this study can be found in online repositories. The names of the repository/repositories and accession number(s) can be found below: The data are part of a private repository at the University of Granada. If you are interested in the dataset, please contact the authors.
